# Co-designing an intervention to prevent rheumatic fever in Pacific People in South Auckland: a study protocol

**DOI:** 10.1186/s12939-022-01701-9

**Published:** 2022-07-21

**Authors:** Siobhan Tu’akoi, Malakai Ofanoa, Samuela Ofanoa, Hinamaha Lutui, Maryann Heather, Rawiri McKree Jansen, Bert van der Werf, Felicity Goodyear-Smith

**Affiliations:** 1 Pacific Health Section, Faculty of Medical and Health Sciences, University of Auckland, PB 92019, Auckland, 1142 New Zealand; 2 Alliance Health Plus, Mount Wellington, Auckland, New Zealand; 3 National Hauora Coalition, Auckland, New Zealand; 4 Epidemiology and Biostatistics, University of Auckland, Auckland, New Zealand; 5 Department of General Practice and Primary Health Care, University of Auckland, Auckland, New Zealand

**Keywords:** Rheumatic fever, Pasifika, Pacific, Co-design, Participatory, Community, Fa’afaletui

## Abstract

**Background:**

Rheumatic fever is an autoimmune condition that occurs in response to an untreated Group A Streptococcus throat or skin infection. Recurrent episodes of rheumatic fever can cause permanent damage to heart valves, heart failure and even death. Māori and Pacific people in Aotearoa New Zealand experience some of the highest rates globally, with Pacific children 80 times more likely to be hospitalised for rheumatic fever and Māori children 36 times more likely than non-Māori, non-Pacific children. Community members from the Pacific People’s Health Advisory Group, research officers from the Pacific Practice-Based Research Network and University of Auckland researchers identified key health priorities within the South Auckland community that needed to be addressed, one of which was rheumatic fever. The study outlined in this protocol aims to co-design, implement, and evaluate a novel intervention to reduce rheumatic fever rates for Pacific communities in South Auckland.

**Methods:**

This participatory mixed-methods study utilises the Fa’afaletui method and follows a three-phase approach. Phase 1 comprises a quantitative analysis of the rheumatic fever burden within Auckland and across New Zealand over the last five years, including sub-analyses by ethnicity. Phase 2 will include co-design workshops with Pacific community members, families affected by rheumatic fever, health professionals, and other stakeholders in order to develop a novel intervention to reduce rheumatic fever in South Auckland. Phase 3 comprises the implementation and evaluation of the intervention.

**Discussion:**

This study aims to reduce the inequitable rheumatic fever burden faced by Pacific communities in South Auckland via a community-based participatory research approach. The final intervention may guide approaches in other settings or regions that also experience high rates of rheumatic fever. Additionally, Māori have the second-highest incidence rates of rheumatic fever of all ethnic groups, thus community-led approaches ‘by Māori for Māori’ are also necessary.

**Trial registration:**

The Australian New Zealand Clinical Trial Registry has approved the proposed study: ACTRN12622000565741 and ACTRN12622000572763.

**Supplementary Information:**

The online version contains supplementary material available at 10.1186/s12939-022-01701-9.

## Background

Rheumatic fever is an autoimmune condition that occurs in response to an untreated Group A streptococcal (GAS) throat or skin infection, primarily affecting children aged 4–19 years [1]. Recurrent episodes of rheumatic fever can lead to rheumatic heart disease, resulting in heart valve damage, heart failure and even death. Although cases have steadily declined across most developed countries, high incidence rates continue to persist for Indigenous and Pacific populations across Aotearoa New Zealand (NZ) and Australia [2]. In an analysis of hospitalisation data from 2000–2018, Māori and Pacific people in NZ comprised 92.6% of acute rheumatic fever cases below the age of 30 years [3]. Among 5–14-year-olds, Pacific children were 80 times more likely to develop acute rheumatic fever and Māori children were 36 times more likely compared to non-Māori non-Pacific children [3]. Within the 20 District Health Boards (DHBs) covering specific geographical areas in New Zealand, Counties Manukau DHB serves South Auckland, including a high proportion of the Pacific population [4]. Since at least 2009, Counties Manukau DHB has had the highest incidence rates nationally, reporting first episodes of rheumatic fever at 14.7 per 100,000 in 2018 [5].

Factors contributing to these health inequities have previously been identified including socio-economic deprivation, inadequate access to healthcare, racism, negative experiences with healthcare professionals, overcrowding, low health literacy and genetic susceptibility [5, 6]. In 2011, the Ministry of Health invested approximately NZD 65 million in the multifaceted ‘Rheumatic Fever Prevention Programme’, aimed at reducing national rheumatic fever rates via mass media awareness campaigns, sore throat swabbing services, healthy homes referrals and enhanced clinical tools and training for health professionals [7]. As part of this programme, the NZ Government set a target in 2012 to reduce national incidence rates to 1.4 per 100,000 by 2017, however, this was not achieved. A range of interventions have been implemented over the years, including the school-based throat swabbing programme ‘Mana Kidz’ led by the National Hauora Coalition (a Māori primary health organisation). This nurse-led programme identifies and treats superficial GAS infections in South Auckland schools and has contributed to stabilising rates for Māori [5, 8]. However, inequities still exist and there is a need for a ‘by Pacific people, for Pacific people’ approach in the community.

The Pacific People’s Health Advisory Group (PPHAG) and the Pacific Practice-Based Research Network (PPBRN) are working together to address such health inequities faced by Pacific communities. PPHAG was set up after a South Auckland-based general practitioner and a Pacific patient attended a patient and clinician engagement programme in North America, focused on the importance of co-design and empowering communities to engage in research [9]. PPHAG is comprised of South Auckland community members from a range of age groups, Pacific ethnicities and professions and aims to identify where research is most needed for Pacific communities in South Auckland. PPBRN was established through Alliance Health Plus, a Pacific-led Primary Health Organisation (PHO). Through the PHO, each general practice designated a staff member to act as a research officer, such as a general practitioner, nurse, or manager [9]. Senior researchers from the University of Auckland, with considerable expertise in Pacific health and primary health care research, then collaborated with PPHAG and PPBRN to provide training on different Pacific methodologies and how to ask meaningful research questions. What resulted from this process was a series of priority research questions developed by the community members and practices. The first research priority related to ensuring Pacific people could access and take medication to prevent gout, and we have previously published a protocol paper for this research project [10]. The second research priority identified was reducing the inequitable rheumatic fever burden faced by Pacific communities, particularly in South Auckland. PPHAG and PPBRN recognise the devastating outcomes rheumatic fever and rheumatic heart disease has on their own families, communities, and Pacific people in general.

With a research question set by the community, this project is based on co-design and community-based participatory research principles. This approach de-centralises research expertise and recognises the knowledge of community members as equally valid and critical throughout each stage of the research in order to achieve social change [11]. Ensuring the project is led by the community for which outcomes are intended is important and also can ensure relevancy and acceptability of the final intervention. This paper presents a protocol for a co-designed novel intervention led by Pacific community members, clinicians and general practice staff, and researchers.

## Methods/design

### Aims and objectives

This study aims to co-develop, implement, and evaluate an innovative intervention to reduce rates within Pacific communities. The objectives are:

1. To determine the burden of GAS infections, acute rheumatic fever, and rheumatic heart disease in Auckland general practices, comparing Pacific, Māori, and non-Pacific non-Māori groups over the last five years.
2. To co-design a novel approach to prevent GAS infections progressing to rheumatic fever within Pacific communities in South Auckland.
3. To evaluate the implementation and effectiveness of the co-designed intervention using an implementation science approach.
4. To create an implementation framework that can guide future implementation roll-out within other settings in NZ.

### Study design

This mixed methods study utilises the *Fa’afaletui* paradigm. Traditionally a Samoan conversational practice relating to serious discussions, *fa’afaletui* was first introduced as a research method by Tamasese and colleagues as a way of facilitating the gathering and critical validation of different knowledge types [12, 13]. It centres on *fa’a*, the ways of sharing and validating knowledge from different groups or *fale* (houses), and *tui*, weaving these together to reach a consensus [14]. Tuia and Cobb emphasise that understanding the cultural practices and social structures in a traditional *fa’afaletui* can strengthen its use as a research method [13]. To discuss important issues within the family and wider village, each Samoan family will meet, allowing for the *matai* (chief) to hear all perspectives. The *fa’afaletui a matai*, which refers to a highly important meeting of the chiefs, can then be described from three levels: top of the mountain, top of the tree and from the canoe. The ‘top of the mountain’ perspective relates to the high chief who opens and closes the meeting, the ‘top of the tree’ perspective relates to the chief orator who presents discussion topics and provides opportunities for *matai* to present, and the ‘persons in the canoe’ represent the *matai* who bring forth the voices and perspectives of their families [13]. Collective decision-making and mutual respect are key principles both in a traditional *fa’afaletui* and when applied in a research sense. The method allows for researchers and participants to work collaboratively together towards a shared goal, ensuring that all voices, opinions, and conflicting perspectives on a serious topic are discussed in a respectful manner. In this project, we consider the *fa’afaletui* approach for the issue of rheumatic fever and how different perspectives and data sources can help to achieve positive outcomes. This will include a national overview of the issue (view from the top of the mountain), a regional outlook (top of the tree) and local community perspectives such as community members, patients, and primary care clinicians (people in the canoe).

The intervention that is co-designed by groups will be informed by a stocktake of current and past interventions as part of a broad scoping review, focusing on Pacific and Māori populations. Interventions will be charted to compare the type of initiative (e.g., awareness, throat swabbing), who is leading it (e.g., nurse, iwi, health organisation) and the outcomes it has had in relation to rheumatic fever.

#### Phase 1: Quantitative assessment of rheumatic fever burden

Phase 1 will be focused on a quantitative assessment of rheumatic fever burden nationally, regionally and for different sub-groups. Observational time series will be used to determine the incidence of GAS infections, acute rheumatic fever cases and related hospitalisations over a five-year period. We also wish to identify what medication is prescribed after a diagnosis and whether this is adhered to for the appropriate length of time. Progressions from rheumatic fever to rheumatic heart disease will also be explored.

To explore these trends, we will use secondary anonymised data from the National Minimum Dataset, the national collection of hospital discharge information [15]. This overview will include incidence rates of first episode rheumatic fever hospitalisations for each year nationally, by DHB and by prioritised ethnic group. To further explore the burden in Auckland, we will be requesting de-identified, routinely collected clinical data from four PHOs (Alliance Health Plus, National Hauora Coalition, ProCare and Tamaki Health) which serve the majority of Pacific and Māori populations in Auckland. Alliance Health Plus is a Pacific-led PHO with approximately 120,000 enrolled patients (28% Pacific, 12% Māori) across 40 general practices while the National Hauora Coalition, a Māori-led PHO, serves around 136,000 patients (14% Pacific, 14% Māori) in 26 clinics [16]. ProCare is a large PHO supporting almost 800,000 patients across Auckland (12.7% Pacific and 9.6% Māori) while Tamaki Health is based in South Auckland and serves 230,000 enrolled patients (33% Pacific and 16% Māori) across 45 general practices [16].

De-identified data will be requested from the PHOs including demographic variables, numbers of sore throat or skin swabs taken, numbers of GAS positive swabs positive, rheumatic fever diagnoses, rheumatic heart disease diagnoses, and related medication or treatment pathways (see supplement for full overview). The incidence rate denominator will include all patients enrolled in the four PHOs at the point of data extraction. De-identified data will be transferred into a password protected file within a secure University of Auckland drive using an encrypted memory stick. Data will be analysed in R and descriptive statistics used to explore the incidence of GAS positive swabs and rheumatic fever/heart disease cases by ethnicity, age group, year, and deprivation level for 2022, 2021, 2020, 2019 and 2018. We will also explore what proportion of patients accessed prescriptions and associated medications. Differences between ethnic groups will be estimated using a generalised linear mixed models with binomial or Poisson distribution. Phase 1 of this project will provide a clear overview of the burden of rheumatic fever in NZ and for Pacific and Māori.

#### Phase 2: Co-designing and implementing a novel intervention

Phase 2 of this project will include a stocktake of programmes and interventions aimed at preventing GAS infections and progressions to acute rheumatic fever in NZ, focusing in particular on Pacific and Māori populations. This will occur as part of a scoping review and build on previous reviews [17, 18] to explore the range of approaches, whether they have been successful, and the implications for Pacific and Māori communities. The stocktake will include the Ministry of Health’s ‘Rheumatic Fever Prevention Programme’ initiated by the NZ Government in 2011, and many other initiatives including school-based throat swabbing programmes such as Mana Kidz and awareness campaigns at a local community level [7, 8]. Concise summaries of the scoping review results will be produced using a variety of techniques such as PowerPoint presentations, brochures, posters and storyboards. This will aid in informing workshop participants of what has been tried before, what has been successful and what might be useful for the design and delivery of the intervention. Participants will also have an opportunity to consider the known environmental risk factors linked with acute rheumatic fever such as family history, household crowding and damp and mouldy housing.

A series of day-long workshops will be conducted with PPHAG, PPBRN and other relevant Pacific community members and stakeholders to explore their views of current and past rheumatic fever interventions and to co-design alternatives. Participant information sheets and consent forms which provide a background of the study, why it is being conducted and what participation includes will be provided to all participants. Participation is completely voluntary. Consent forms will need to be signed and returned to the research team either in person or via email for online workshops before participation in workshops can occur. Upon learning together about data trends and previous rheumatic fever interventions, participants will then collaboratively workshop innovative solutions using their expertise as members of the Pacific community in South Auckland. Groups may be provided with large sheets of paper and whiteboards to brainstorm, and these data will be collected at the end of the workshop. Thematic analysis of data using NVivo software will utilise a general inductive approach to ensure the development of the intervention comes from the data rather than preconceived theories or ideas [19]. Once an intervention has been workshopped, a group of advisors including Pacific and Māori experts in rheumatic fever will help refine it.

Where possible, workshops will take place in-person (or virtually depending on COVID-19 restrictions) using cultural protocols and approaches. Dialogue within the workshops will follow Talanga, a Tongan participant-centred approach that means “interactive talking with a purpose” and enables empowering and interactive dialogue towards action [20]. Throughout the series of workshops, data will be analysed and synthesised to be presented back to the participants. This ensures participants are respected as owners of their data and provides an opportunity to review the data collected and how it has been analysed. The final co-designed intervention will be informed by the Fonofale model which outlines the core pillars of Pacific ways of life including physical, spiritual, mental and overall wellbeing, while being grounded by family and supported by the cultural values of community, collectiveness and reciprocal relationships [21].

In order to map the implementation of the final intervention, a framework will be developed using a logic model of change [22, 23]. This will outline the inputs (such as resources, personnel and funding required), activities (encompassing the tasks necessary for implementation), outputs (measures demonstrating implementation has occurred) and short-term outcomes (the expected changes).

#### Phase 3: Evaluating the implementation of the intervention

Phase 3 will comprise an evaluation of the implementation of the co-designed intervention. The exact details of the evaluation including the type of evaluation, study participants and duration will depend on the nature of the intervention constructed from Phase 2. Study participants may include patients with rheumatic fever, their families, health professionals and/or the wider community. Overall, the evaluation will likely focus on:

1. *Process:* This refers to the implementation, delivery, and adaptation of intervention components to achieve the desired outcome. For this project, measures will assess the implementation of the intervention in terms of how feasible it is, its acceptability to the intended population (for example Pacific rheumatic fever patients, families, and communities) and its accessibility. In-depth interviews and focus groups may be undertaken with these groups to explore their experiences and use of the intervention and discuss any access barriers or opportunities for improvement. During this process, the Fonofale model will assess how successfully the intervention has connected with the physical, mental, spiritual, and cultural needs of participants. Further data may be collected from the personnel administering the intervention, via surveys, interviews or focus groups.
2. *Mediators of change*: This includes assessing whether the intervention is addressing perceived barriers or enhancing enablers. Depending on the nature of the intervention, this may include collecting data on the frequency and duration of the intervention, how much it costs and the events or resources that influenced its delivery. Changes to the delivery of the intervention may occur in response to feedback and process data analyses.
3. *Outcomes:* This refers to how effective the intervention is at achieving the intended outcomes. While the long-term goal for this study is to reduce the incidence of acute rheumatic fever for Pacific populations, the short-term outcomes that may be assessed will depend on the nature of the intervention, for example, increasing awareness of rheumatic fever in Pacific families or improving accessibility of related healthcare and medications. Data collected here may include surveys, interviews or focus groups, patient data on rheumatic fever diagnoses, uptake of prescriptions, and/or hospitalisation data.

The evaluation framework RE-AIM (reach, effectiveness, adoption, implementation and maintenance) will be used to guide analyses and evaluate the impact of the intervention within the community [24]. In order to achieve the outcomes in this study and enhance knowledge translation, we will engage with all relevant stakeholders to explore the impact on the intervention in the real-world setting. We acknowledge that although the logic model and stepwise plan presented are linear and straightforward, the implementation in reality may be complex and impacted by a variety of external factors. An iterative feedback loop with providers, patients, families, and the wider community is therefore important to ensure that challenges are being identified, addressed and adapted to.

In order to guide the implementation of the intervention for other settings, a framework based on the Consolidated Framework for Implementation Research model will be developed [25]. This model consolidates key constructs from a range of implementation theories into five core domains: intervention characteristics, process, individuals involved, inner setting and outer setting [25]. These provide a system for synthesising and building knowledge about what approaches work where, while assessing the potential barriers and enablers in a particular setting. This model will help to guide a framework for adapting this intervention for use in other settings.

## Discussion

First and foremost, this study aims to reduce health inequities faced by Pacific populations in NZ related to rheumatic fever and rheumatic heart disease. Pacific people consistently experience the highest incidence rates of rheumatic fever in NZ among all ethnic groups which is influenced by a range of complex factors such as housing, racism, access to health care, and health literacy [5]. It will be important to ensure that the co-designed intervention acknowledges the historical and structural factors that have maintained inequities to date and does not create further stigma or blame for the affected communities [26]. Addressing rheumatic fever and rheumatic heart disease rates will not only improve health outcomes for Pacific families and communities but will also have long-term benefits for reducing hospitalisations and health system related costs in NZ.

Knowledge created from this study may be translated to guide approaches in other settings and populations. Māori populations experience similar inequitable outcomes related to rheumatic fever with the second highest incidence rates in NZ. Interventions which adopt a Kaupapa Māori ‘by Māori for Māori’ approach have shown success and ensure that healthcare is delivered equitably and appropriately [5]. In addition to DHBs within the Auckland region, hospitalisation rates for first episodes of rheumatic fever are also high in Northland, Waikato, Lakes and Hawkes Bay [15]. Community-led approaches are needed in each context to ensure interventions are appropriate, relevant, and effective for the local community. The participatory research process outlined in this protocol empowers the community to set research directions from the beginning, ensuring that projects are relevant, needed in the community and can lead to better health outcomes.

## Supplementary Information

**Additional file 1:** **Supplementary Material: **Variables to be obtained for individual visit data from the Primary Health Organisations.

## Data Availability

Not applicable.

## Abbreviations

DHB: District Health Board
NZ: Aotearoa New Zealand
PHO: Primary Health Organisation
PPBRN: Pacific Practice-Based Research Network
PPHAG: Pacific People’s Health Advisory Group
