# Supplementary material for: Co-designing an intervention to prevent rheumatic fever in Pacific People in South Auckland: a study protocol
Source: Int J Equity Health. 2022 Jul 21;21:101. doi: 10.1186/s12939-022-01701-9 (PMC9302560; doi:10.1186/s12939-022-01701-9)
Supplement: Supplementary file 1 — Additional file 1: Supplementary Material: Variables to be obtained for individual visit data from the Primary Health Organisations. [file 12939_2022_1701_MOESM1_ESM.docx]

**Supplementary Material: Variables to be obtained for individual visit data from the Primary Health Organisations**

- Patient identifier - [The primary health organisation will de-identify data and apply codes to replace the National Health Index number].
- Sex
- Date of birth
- Ethnicity - prioritised
  - Total Pacific (as a binary variable – any Pacific ethnicity reported Y/N)
  - Total Tokelauan (ethnicity group Level 2; binary)
  - Total Niuean (ethnicity group Level 2; binary)
  - Total Tongan (ethnicity group Level 2; binary)
  - Total Cook Island Māori (ethnicity group Level 2; binary)
  - Total Samoan (ethnicity group Level 2; binary)
  - Total Other Pacific Peoples (ethnicity group Level 2; binary)
  - Total Pacific Peoples not further defined (ethnicity group Level 2; binary)
  - Total Fijian (ethnicity group Level 4; binary)
  - Māori (ethnicity group Level 1; binary)
  - European (ethnicity group Level 1; binary)
  - Asian (ethnicity group Level 1; binary)
  - MELAA (ethnicity group Level 1; binary)
- NZ Deprivation (NZDep) index decile
- Overall numbers of patients enrolled (and by ethnicity)

From the period of 31 March 2017 to 1 April 2022:

- Numbers of throat or skin swabs taken to test for Group A streptococcus
- Numbers of throat or skin swabs positive for Group A streptococcus
- Within cases of positive Group A streptococcus, what was the treatment/management (i.e. amoxicillin)
- All notifications of acute rheumatic fever cases
- Dates of rheumatic fever diagnoses
- All cases of rheumatic heart disease
- Numbers and dates of rheumatic fever-related prescriptions (e.g. penicillin)
- Prescribed treatment dose and route of administration
- Hospital admission(s) with the primary diagnosis of rheumatic fever
- Hospital admission(s) with the primary diagnosis of rheumatic heart disease
- Date of death if applicable (with a rheumatic fever or rheumatic heart disease diagnosis)
